# Lack of Association between NADPH Quinone Oxidoreductase 1 (NQO1) Gene C609T Polymorphism and Lung Cancer: A Case-Control Study and a Meta-Analysis

**DOI:** 10.1371/journal.pone.0047939

**Published:** 2012-10-24

**Authors:** Shujie Guo, Min Gao, Xiaobo Li, Yuqiong Li, Shaoli Chu, Dingliang Zhu, Wenquan Niu

**Affiliations:** 1 State Key Laboratory of Medical Genomics, Ruijin Hospital, Shanghai Jiao Tong University School of Medicine, Shanghai, China; 2 Department of Respiratory, The Fourth Affiliated Hospital of Harbin Medical University, Harbin, Heilongjiang, China; 3 Department of Hypertension, Ruijin Hospital, Shanghai Jiao Tong University School of Medicine, Shanghai, China; Kyushu University, Japan

## Abstract

**Background:**

The association between NAD(P)H:quinone oxidoreductase 1 (*NQO1*) gene C609T polymorphism (rs1800566) and lung cancer has been widely evaluated, and a definitive answer so far is lacking. We first conducted a case-control study to assess this association in northeastern Han Chinese, and then performed a meta-analysis to further address this issue.

**Methodology/Principal Findings:**

This case-control study involved 684 patients clinically diagnosed as lung cancer and 602 age-matched cancer-free controls from Harbin city, Heilongjiang province, China. Genotyping was conducted using the PCR-LDR (ligase detection reactions) method. Meta-analysis was managed by STATA software. Data and study quality were assessed in duplicate. Our case-control association study indicated no significant difference in the genotype and allele distributions of C609T polymorphism between lung cancer patients and controls, consistent with the results of the further meta-analysis involving 7286 patients and 9167 controls under both allelic (odds ratio (OR) = 0.99; 95% confidence interval (CI): 0.92–1.06; P = 0.692) and dominant (OR = 0.98; 95% CI: 0.89–1.08; P = 0.637) models. However, there was moderate evidence of between-study heterogeneity and low probability of publication bias. Further subgroup analyses by ethnicity, source of controls and sample size detected no positive associations in this meta-analysis.

**Conclusions:**

Our study in northeastern Han Chinese, along with the meta-analysis, failed to confirm the association of *NQO1* gene C609T polymorphism with lung cancer risk, even across different ethnic populations.

## Introduction

Lung cancer is one of the common malignancies, and nearly 1.3 million new cases are diagnosed annually worldwide [1], [2]. Evidence is mounting suggesting that the cause of lung cancer is multifactorial, and part is due to genetic defects. In the last decade, exhaustive efforts have been devoted to unraveling the genetic underpinnings of lung cancer; however, its driving genes and genetic determinants that attribute to the development of lung cancer so far remain elusive.

The gene encoding NAD(P)H:quinone oxidoreductase 1 (*NQO1*) is a promising candidate in the pathogenesis of lung cancer [3], [4]. NQO1 is a cytosolic enzyme, and catalyzes the reduction of two electrons of quinoid compounds to generate the less-toxic hydroquinones, which can alleviate cancer carcinogenesis [5], [6]. In tissues of human lung cancer, *NQO1* gene was observed to be over-expressed [5], [7]–[9]. It is therefore of added interest to identity genetic defects of *NQO1* gene responsible for its enzyme activity, further accountable for lung carcinogenesis. The transition of C to T at position 609 (rs1800566) of *NQO1* gene can lead to reduced activity of quinone reductase [10]–[12]. The C609T polymorphism has been widely evaluated in association with lung cancer across various ethinicities, yet with conflicting results, possibly due to the insufficient sample sizes, genetic backgrounds, and selection of study populations.

In this study, we first decided to assess the assocation of C609T polymorphism of *NQO1* gene with lung cancer risk in a large northeastern Han Chinese population. Then, given the accumulating data and to shed some light on current uncertain claims, we sought to conduct a comprehensive meta-analysis of this association from both English and Chinese literature.

**Table 1:** The baseline characteristics of our study population in case-control study.

Variables	Patients (n = 684)	Controls (n = 602)	P*
**Age (years)**	57.24 (9.84)	56.80 (9.95)	0.776
**Sex (male, %)**	72.78	66.49	0.013
**Smoking (%)**			<0.005
Current	28.22	6.99	
Ever	8.04	0.54	
None	63.74	92.47	
**Drinking (%)**			<0.005
Current	15.23	5.38	
Ever	1.61	2.69	
None	83.16	91.94	
**Lung cancer type (%)**			
Squamous cell cancer	32.26	NA	
Adenocarcinoma	37.54	NA	
Small cell cancer	20.83	NA	
Unspecified	9.38	NA	

*Abbreviations:* NA, not available. Data are expressed as mean (standard deviation or SD) or percentage as indicated.

* P values were calculated by using unpaired t-test for age, and by χ^2^ test for other category variables.

**Table 2:** The alleles and genotype distributions of *NQO1* gene C609T polymorphism between cases (n = 682) and controls (n = 597).

Status	C609T genotypes (number)			C609T alleles (%)	
	CC	CT	TT	C	T
**Cases**	187	327	168	51.39	48.61
**Controls**	171	282	144	52.26	47.74
	?^2^ = 0.2385; P = 0.888			?^2^ = 0.1923; P = 0.661	
Additive mode**l** a		Dominant model^a^		Recessive model^a^	
0.99; 0.85–1.17; 0.634		1.01; 0.81–1.33; 0.607		1.04; 0.80–1.34; 0.791	

*P*-values were calculated using χ^2^-test from a series of 3×2 contingency tables for genotype data and 2×2 contingency tables for allele data.

a Data are expressed as odds ratio; 95% confidence interval; *P*-values for genetic modes of inheritance.

## Methods

### Study Population

The design of this study has been described previously [13]. Briefly, this was a hospital-based case-control study with a total of 1286 subjects consecutively recruiting from three hospitals in Harbin city, Heilongjiang province, China. The study population included 684 patients clinically diagnosed as lung cancer and 602 age-matched cancer-free controls, and all subjects were local residents of Han descent. This study was approved by the Ethics Committee of Harbin Medical University, and was conducted according to the Declaration of Helsinki Principles. All subjects signed the written informed consent.

**Figure 1:**
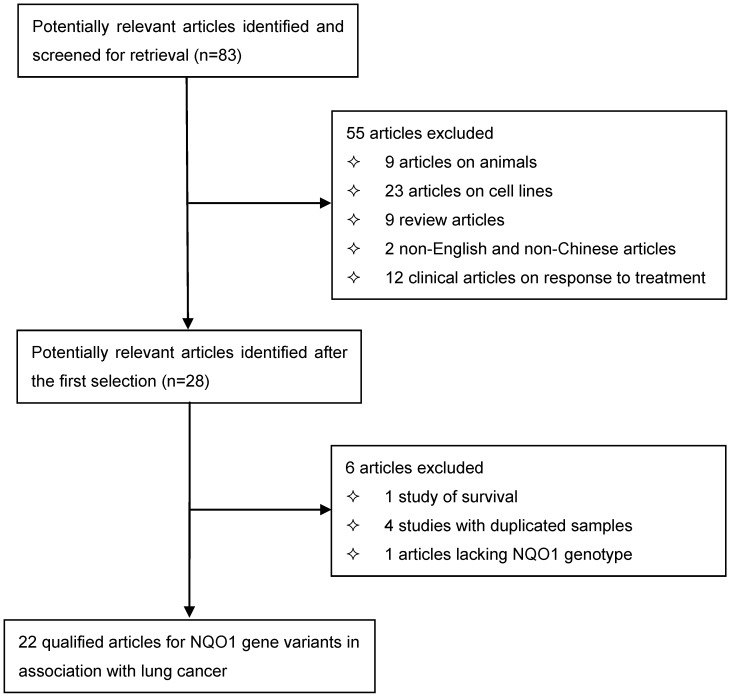
Flow diagram of search strategy and study selection.

### Diagnostic Criteria and Demographic Characteristics

Lung cancer was diagnosed by chest radiograph and either high resolution computed tomography (CT) or enhanced CT or positron emission computed tomography (PET)-CT scan, which was confirmed by clinical doctors of respiratory medicine. Those who were susceptible to lung cancer were further pathologically confirmed by biopsy, and those with normal CT or enhanced CT or PET-CT results were treated as cancer-free controls in this study. Clinical subtypes of lung cancer included squamous cell cancer, adenocarcinoma, small cell cancer and unspecified lung cancer. Age and gender were recorded at enrollment. The status of cigarette smoking and alcohol drinking were defined at the time of the survey. Smoking was expressed by percent of ever or current smoking. Drinking was categorized as never, ever or current drinking. Here, current drinking referred to consumption of at least one alcoholic drink during the past 30 days.

**Table 3:** The baseline characteristics of all qualified studies in this meta-analysis.

Author	Disease type	Match	Ethnicity	Design	Age, years	
					Cases	Controls
Wiencke JK et al.	Lung cancer	age, sex, race	Mixed	Population	62.10	62.20
Wiencke JK et al.	Lung cancer	age, sex, race	African-American	Population	62.10	62.20
Chen H et al.	Lung cancer	age, sex, race, smoking,	Asian	Population	64.50	65.10
Chen H et al.	Lung cancer	age, sex, race, smoking,	Caucasian	Population	64.50	65.10
Chen H et al.	Lung cancer	age, sex, race, smoking,	Mixed	Population	64.50	65.10
Lin YH et al.	Lung cancer	non-match	Asian	Hospital	NA	NA
Lewis SJ et al.	Lung cancer	age, sex, smoking	Caucasian	Hospital	67.4	59.5
Xu LL et al.	Lung cancer	age, sex, smoking	Caucasian	Hospital	NA	NA
Xu LL et al.	Lung cancer	age, sex, smoking	Mixed	Hospital	NA	NA
Yin L et al.	Lung cancer	age, sex, race	Asian	Hospital	60.30	60.90
Sunaga N et al.	Lung adenocarcinoma	age, sex, race	Asian	Hospital	63.00	65.00
Hamajima N et al.	Lung cancer	NA	Asian	Hospital	NA	NA
Lin P et al.	Lung cancer	sex	Asian	Hospital	64.00	58.00
Alexandrie AK et al.	Lung cancer	NA	Caucasian	Population	66.00	44.00
Lan Q et al.	Lung cancer	age, sex	Asian	Population	NA	NA
Liang GY et al.	Lung cancer	age, sex	Asian	Hospital	60.90	60.50
Bock CH et al.	Lung cancer	age, race	Caucasian	Population	NA	NA
Bock CH et al.	Lung cancer	age, race	African-American	Population	NA	NA
Chan EC et al.	Lung cancer	age	Asian	Hospital	62.65	61.40
Lawson KA et al.	Lung cancer	NA	Caucasian	Population		
Saldivar SJ et al.	Lung cancer	age, sex, race, smoking	Caucasian	Population	61.55	61.45
Saldivar SJ et al.	Lung cancer	age, sex, race, smoking	African-American	Population	61.55	61.45
Saldivar SJ et al.	Lung cancer	age, sex, race, smoking	Caucasian	Population	61.55	61.45
Skuladottir H et al.	Lung cancer	age, sex, study	Caucasian	Population	NA	NA
Sorensen M et al.	Lung cancer	age, sex, smoking	Caucasian	Population	NA	NA
Yang M et al.	Lung cancer	age, sex, smoking, al	Asian	Hospital	55.40	48.30
Cote ML et al.	NSCLC	age, race, BMI, smoking	Caucasian	Population	60.30	59.50
Cote ML et al.	NSCLC	age, race, BMI, smoking	African-American	Population	57.40	57.50
Eom SY et al.	Lung cancer	age, sex, smoking	Asian	Hospital	63.90	62.60
Timofeeva M et al.	Lung cancer	age, sex	Caucasian	Population	NA	NA
Guo S et al. (the present study)	Lung cancer	age, sex, smoking	Asian	Hospital	57.24	56.80

*Abbreviations*: NA, not available.

**Table 4:** The baseline characteristics of all qualified studies in this meta-analysis (Continued).

Author	Gender (Males, %)		Smoking status (%)		Score	Cases	Controls
	Cases	Controls	Cases	Controls		CC/CT/TT	CC/CT/TT
Wiencke JK et al.	0.763	0.697	current/former/never: 45.90/47.54/6.56	27.95/26.09/45.96	6	29/32*	52/109*
Wiencke JK et al.	0.763	0.697	current/former/never: 64.66/32.76/2.59	32.35/27.94/39.71	6	77/39*	83/53*
Chen H et al.	NA	NA	NA	NA	7	54/48/7	64/78/25
Chen H et al.	NA	NA	NA	NA	7	81/49/5	105/62/4
Chen H et al.	NA	NA	NA	NA	7	61/18/4	60/39/3
Lin YH et al.	0.778	0.445	NA	NA	2	12/63/20	41/73/22
Lewis SJ et al.	0.638	0.539	current/former/never: 29.8/68.1/2.1	25.5/48.5/26.1	7	56/24/2	111/32/2
Xu LL et al.	0.547	0.451	current/former/never: 40.79/53.07/6.14	18.81/45.90/35.29	7	513/246/21	715/341/40
Xu LL et al.	NA	NA	current/former/never: 40.79/53.07/6.14	18.81/45.90/35.29	7	18/14/2	20/6/1
Yin L et al.	0.726	0.726	smoing/non-smoing: 53.6/46.4	53.6/46.4	7	28/39/17	26/41/17
Sunaga N et al.	0.626	0.711	smoker: 62.12	66.43	8	83/93/22	52/77/23
Hamajima N et al.	0.599	0.439	NA	NA	6	87/71/34	154/179/66
Lin P et al.	0.722	0.684	current/former/never: 48.0/9.6/42.4	39.2/7.8/53.0	7	57/141/0	95/237/0
Alexandrie AK et al.	0.221	0.344	ever/never/no information: 59.5/5.9/34.6	51.5/43.8/4.7	7	345/168/11	368/153/9
Lan Q et al.	0.660	0.650	smoing/non-smoing: 6/94	7/93	8	37/57/25	32/54/23
Liang GY et al.	0.704	NA	NA	NA	2	37/79/36	53/71/28
Bock CH et al.	0.416	0.497	never	never	10	93/37*	87/57*
Bock CH et al.	0.416	0.497	never	never	10	21/10*	21/8*
Chan EC et al.	0.827	0.858	NA	NA	6	25/37/13	45/83/34
Lawson KA et al.	1.000	1.000	100	100	10	244/109*	243/117*
Saldivar SJ et al.	0.551	0.551	current/former/never: 17.07/56.17/26.76	17.07/54.12/28.81	9	454/205/24	480/186/17
Saldivar SJ et al.	0.551	0.551	current/former/never: 17.07/56.17/26.76	17.07/54.12/28.81	9	15/17/4	15/14/7
Saldivar SJ et al.	0.551	0.551	current/former/never: 17.07/56.17/26.76	17.07/54.12/28.81	9	67/33/7	69/35/3
Skuladottir H et al.	0.540	0.530	former 96.1	64.8	7	108/45*	227/119*
Sorensen M et al.	NA	NA	current/former/never: 84/13.3/2.7	74.7/21.9/3.3	10	162/83/9	176/80/11
Yang M et al.	0.676	0.618	cuurent/former/never: 43.1/22.0/34.9	35.1/15.0/45.3	7	110/158/46	120/166/61
Cote ML et al.	0.000	0.000	current/former/never: 57.6/34.2/8.2	17.1/32.3/50.6	10	271/97/19	271/119/15
Cote ML et al.	0.000	0.000	current/former/never: 70.4/22.6/7.0	25.8/28.3/45.9	10	77/32/4	79/36/6
Eom SY et al.	0.767	0.767	current/former/never: 59.9/21.5/18.6	49.9/14.0/36.2	6	122/265*	148/239*
Timofeeva M et al.	0.636	0.629	current/former/never: 75.9/17.3/6.8	36.3/29.0/34.6	6	429/188*	856/411*
Guo S et al. (the present study)	0.728	0.665	current/former/never: 28.22/8.04/93.74	6.99/0.54/92.47	8	187/326/168	172/281/144

*Abbreviations*: NA, not available.

* Data on CC and CT genotypes were provided together.

### Genotyping

Blood samples (1 mL) were collected, and genomic DNA was extracted from white blood cells using the TIANamp Blood DNA Kit (Tiangen Biotect [Beijing] Co., LTD). Genotyping was conducted using the PCR-LDR (ligase detection reactions) method by ABI 9600 system (Applied Biosystems, USA) [14]. Cycling parameters were as the following: 94°C for 2 min; 35 cycles of 94°C for 15 s; 60°C for 15 s; 72°C for 30 s; and a final extension step at 72°C for 5 min. Two specific probes to discriminate the specific bases and one common probe were synthesized (available upon request). The common probe was labeled at the 3′ end with 6-carboxy-fluorescein and phosphorylated at the 5′ end. The reacting conditions of LDR were: 94°C for 2 min, 20 cycles of 94°C for 30 s and 60°C for 3 min. After reaction, 1 mL LDR reaction products were mixed with 1 mL ROX passive reference and 1 mL loading buffer, and then denatured at 95°C for 3 min, and chilled rapidly in ice water. The fluorescent products of LDR were differentiated using ABI sequencer 377 (Applied Biosystems, USA).

**Figure 2:**
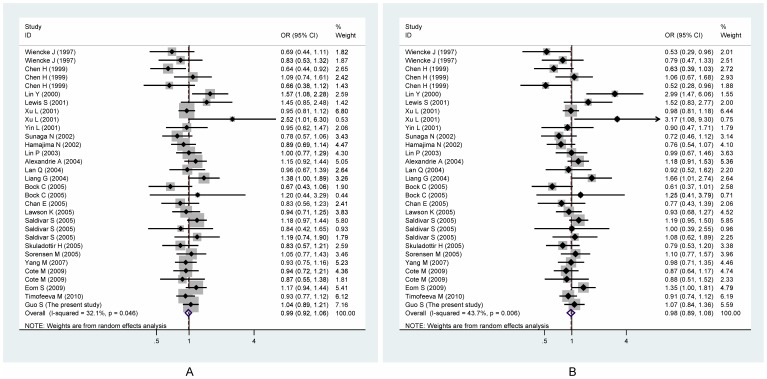
Funnel plots of NQO1 gene C609T polymorphism with lung cancer under both allelic (A) and dominant (B) models.

**Figure 3:**
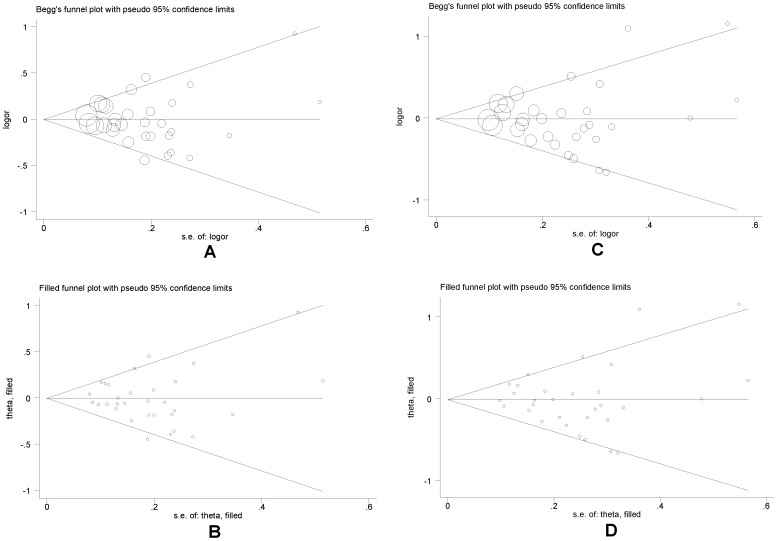
Begg’s and filled funnel plots for studies investigating the effect of NQO1 gene C609T polymorphism on lung cancer under both allelic (A and B) and dominant models (C and D).

### Statistical Analysis

Comparisons between lung cancer patients and controls were conducted by unpaired t-test for continuous variables and by χ^2^ test for categorical variables. To avoid gross genotyping error, C609T polymorphism was checked for consistency with Hardy-Weinberg equilibrium by χ^2^ test. In view of age-matched patients and controls, genotypes were compared by conditional logistic regression analysis under assumptions of additive, dominant and recessive models of inheritance, respectively. Statistical significance was accepted as P<0.05.

### Meta Analysis

This meta-analysis is reported in accordance with the Preferred Reporting Items for Systematic Reviews and Meta-analyses (PRISMA) guideline (please see PRISMA checklist: Table S1) [15].

HuGE Navigator and PubMed, as well as Wanfang database (http://www.wanfangdata.com.cn) were searched up to July 2012 for articles investigating the association between C609T polymorphism and lung cancer risk. The subject terms used were “lung cancer” or “lung neoplasm” and “NAD(P)H:quinone oxidoreductase” or “NQO1”, annexed with “gene” or “allele” or “genotype” or “variant” or “polymorphism” or “mutation”. Articles written in English or Chinese language and studies performed in human subjects were identified. The search result was supplemented by reviews of reference lists for all relevant studies and review articles. In addition, if there were shared or duplicated samples between studies, we recruited those with the large sample size.

Studies were qualified if they met the following criteria: (i) on a retrospective or nested case-control design; (ii) adopt validated genotyping method; (iii) provide genotype counts of *NQO1* gene C609T polymorphism between patients with lung cancer and controls.

Study quality was evaluated by using a quality assessment score explored for genetic association studies by Thakkinstian et al [16]. Total scores ranged from 0 (worst) to 12 (best). The criteria for quality assessment of genetic association between *NQO1* gene C609T polymorphism examined with lung cancer are described in Table S2.

In the meta-analysis, the random effects model using the DerSimonian and Laird method was employed to combine the individual effect size estimates to calculate pooled weighted ORs, and the estimate of heterogeneity was determined using the Mantel-Haenszel model. Between-study heterogeneity was explored by the χ^2^ test, and heterogeneity was assessed by the inconsistency index *I* ^2^ statistic (ranging from 0 to 100%) which is defined as the percentage of the observed between-study variability that is due to heterogeneity rather than to random error [17].

Publication bias was assessed by the Begg’s funnel plot, Egger’s test and the trim and fill method [18]. For the *I* ^2^ and Egger’s statistics, statistical significance was set at 0.1. All statistical analyses were managed by STATA software (version 11.0).

## Results

### Baseline Characteristics

The demographics and risk factors of the study population are summarized in Table 1. Cases and controls were well matched by age. Male gender, smoking and drinking were associated with increased risk for lung cancer. Among all lung cancer patients, the subtype of adenocarcinoma, squamous cell cancer, small cell cancer, and unspecified cancer accounted for 37.54%, 32.26%, 20.83%, and 9.38%, respectively.

### Single-locus Analysis

The success rates of genotyping for C609T polymorphism were 99.71% and 99.17% in patients and controls, respectively. Genotype distributions of examined polymorphism respected Hardy-Weinberg equilibrium in controls (P>0.05). There was no significant difference in the genotype and allele distributions of C609T polymorphism between lung cancer patients and controls, and this non-significance was also mirrored under assumptions of the additive (OR = 0.99; 95% CI: 0.85–1.17; P = 0.634), dominant (OR = 1.01; 95% CI: 0.81–1.33; P = 0.607) and recessive (OR = 1.04; 95% CI: 0.80–1.34; P = 0.791) models (Table 2).

### Eligible Articles for Meta-analysis

The initial search yielded 83 potentially relevant articles. After applying the inclusion/exclusion criteria, 22 articles were eligible for inclusion. A flow chart schematizing the process of selecting and excluding articles with specific reasons is shown in Figure 1. The retrieved articles were published between 1997 and 2011, with 20 articles written in English and two in Chinese.

### Study Characteristics

Since more than one study group was included in some articles, we treated them separately. In total, 30 separate studies plus the present study encompassing a total of 7286 patients with lung cancer and 9167 controls were finally meta-analyzed, with 12 studies performed in Asians [19]–[29], 12 in Caucasians [19], [30]–[39], 4 in African-Americans [33], [35], [38], [40], and 3 in mixed ethnicities [19], [31], [40]. 18 studies reported matching information on age between patients and controls. The quality score of individual studies ranged from 2 to 10 (mean: 7.6) out of a maximal score of 12. The frequencies of 609T allele varied widely, which were exceedingly higher in Asians than in Caucasians for both patients (28.44 to 54.21% versus 14.23–21.96%) and controls (30.88 to 46.60% versus 12.41–20.47%). Baseline characteristics of qualified studies are shown in Table 3 and Table 4.

**Table 5:** Subgroup analyses of *NQO1* gene 609C/T polymorphism with the risk of lung cancer, and exploration of between-study heterogeneity and publication bias.

Variables	Studies (Cases/Controls), n (n/n)	Allelic model		Dominant model	
		OR (95% CI); P	*I* ^2^ (*P_ϰ2_* ); P_Egger_	OR (95% CI); P	*I* ^2^ (*P_ϰ2_* ); P_Egger_
**Descent of populations**					
Asian	12 (2607/3029)	0.99 (0.88–1.11); 0.879	50.1% (0.024); 0.612	1.01 (0.84–1.22); 0.903	57.9% (0.006); 0.973
Caucasian	12 (4205/5521)	1.01 (0.93–1.09); 0.849	9.4% (0.354); 0.931	1.00 (0.91–1.10); 0.964	14.6% (0.301); 0.834
African-American	4 (296/322)	0.87 (0.66–1.16); 0.341	0.0% (0.932); 0.192	0.88 (0.63–1.23); 0.466	0.0% (0.894); 0.066
Mixed	3 (178/295)	0.94 (0.49–1.80); 0.843	71.1% (0.032); 0.18	0.85 (0.34–2.14); 0.734	78.6% (0.009); 0.057
**Source of controls**					
Population	18 (4011/5141)	0.95 (0.87–1.04); 0.237	18.2% (0.237); 0.06	0.92 (0.82–1.03); 0.136	24.7% (0.163); 0.059
Hospital	13 (3275/4026)	1.04 (0.93–1.15); 0.485	45.5% (0.037); 0.185	1.10 (0.92–1.30); 0.300	57.3% (0.005); 0.180
**Sample size in cases**					
≥500 cases	5 (3288/4178)	1.03 (0.95–1.13); 0.469	17.6% (0.303); 0.963	0.95 (0.83–1.08); 0.419	46.9% (0.005); 0.632
<500 cases	26 (3998/4989)	0.96 (0.88–1.05); 0.398	34.1% (0.047); 0.355	1.04 (0.94–1.16); 0.414	5.9% (0.373); 0.144

Abbreviations: OR, odds ratio; 95% CI, 95% confidence interval.

### Meta-analysis Results

After combining all qualified studies, we found null association of *NQO1* gene C609T polymorphism with lung cancer under both allelic (OR = 0.99; 95% CI: 0.92–1.06; P = 0.692) and dominant (OR = 0.98; 95% CI: 0.89–1.08; P = 0.637) models, and this association suffered from significant evidence of heterogeneity between studies (allelic and dominant models: *I* ^2^ = 32.1% and 43.7%) (Figure 2). However, there was low probability of publication bias for both models (P_Egger_ = 0.608 and 0.81) (Figure 3).

To account for potential sources of heterogeneity, we conducted a set of subgroup analyses according to ethnicity, source of controls and sample size (Table 5). Despite the wide divergence of 609T allele, the risk estimates were comparable in magnitude between Asians and Caucasians. Notably, 609T allele was associated with a 13% reduced, albeit nonsignificant, risk for lung cancer in African-Americans (95% CI: 0.66–1.16; P = 0.341), without heterogeneity (*I* ^2^ = 0.0%) or publication bias (P_Egger_ = 0.192). Upon stratification by source of controls, risk magnitude was relatively stronger in hospital-based studies than in population-based studies, whereas heterogeneity and publication bias tangled the former. Restricting analysis to studies with ≥500 patients still detected no material changes in risk estimates in both allelic and dominant models, indicating the robustness of our results.

## Discussion

Although numerous studies have regarded *NQO1* gene C609T polymorphism as a promising candidate for lung cancer, our case-control study in northeastern Han Chinese, along with the meta-analysis, failed to confirm this relation, even across different ethnic populations. This non-significance was also reflected in larger sample-size studies, which are less prone to chance results, indicating the robustness of our findings. To the authors’ knowledge, this is the most comprehensive meta-analysis investigating the genetic susceptibility of *NQO1* gene C609T polymorphism to lung cancer.

With the increase of genetic association studies, it is highly encouraged to synthesize available data to resolve persistent difficulties in obtaining robust, replicable results. Considering the fact that most common genetic defects usually make a small-to-moderate contribution to future disease risk, this study urges the necessity of very large sample sizes to drive sufficiently precise estimates between genetic variation and disease. Several individual studies have reported positive signals of *NQO1* gene C609T polymorphism with lung cancer [19], [20], [22], [23], [26], [29], [39], [40]; contrastingly, as illustrated in our overall findings among 16453 subjects, there was no detectable risk, even in populations of different descents. However, it is worth noting that the protective effect conferred by 609T allele was appreciably obvious in African-Americans. Considering the relatively few studies were conducted in African-Americans and most of these studies were small, there is an indication of possible benefit, reinforcing additional large studies to confirm or refute this finding. Even though, we cannot rule out a possible significant effect of 609T allele in lung carcinogenesis, and it is also possible that the potential role of the examined polymorphism is diluted or masked by other gene-gene or gene-environment interactions.

Several strengths distinguishing the present investigation merit adequate consideration. First, this is to date the largest synthesis exploring the association of *NQO1* gene C609T polymorphism with lung cancer. Second, the results of the present case-control study were in line with that of the corresponding meta-analysis, and restricting analyses to larger studies generated the similar findings. Third, our results are less prone to selection bias in view of low probability of publication bias.

In addition, some limitations should be considered when interpreting our findings. First, the cross-sectional design of included studies in this meta-analysis may preclude comments on causality, and a survival bias could not be excluded. Second, as with all meta-analyses, publication bias might have occurred because our analyses were based entirely on published studies from English- and Chinese-language journals. Third, although the adopted random-effects model takes both a between-study variance and the within-study variances into account, this model cannot be regarded as a panacea for heterogeneity [41]. Moreover, as stated by Higgins et al [42], the assumption of true quantities from the individual studies following a certain probability distribution in a random-effect model is somewhat arbitrary and makes the interpretation of its predictions difficult. Fourth, data on circulating levels of NQO1 protein or its catalyzed products were unavailable, precluding a more robust assessment of sources of heterogeneity, and making us incapable of comparing their levels across genotypes. Fifth, we focused on only one polymorphism in *NQO1* gene, and did not cover other susceptibility genes or polymorphisms. Given these limitations, we cannot jump to a conclusion until further verification of our findings in vitro, in vivo and in large prospective studies.

To sum up, this case-control study in northeastern Han Chinese, along with the comprehensive meta-analysis, failed to confirm the association of *NQO1* gene C609T polymorphism with lung cancer risk, even across different ethnic populations. Nevertheless, for practical reasons, we hope that this study will not remain just another endpoint of research instead of a beginning to establish the background data to further investigate the molecular mechanisms of *NQO1* gene and lung cancer.

## Supporting Information

Table S1 **Checklist of items to include when reporting a systematic review or meta-analysis (diagnostic review consisting of cohort studies).**
(DOC)

Table S2 **Criteria for quality assessment of genetic association of NQO1 gene C609T polymorphism with lung cancer.**
(DOC)
