# Supplementary material for: Lack of Association between NADPH Quinone Oxidoreductase 1 (NQO1) Gene C609T Polymorphism and Lung Cancer: A Case-Control Study and a Meta-Analysis
Source: PLoS One. 2012 Oct 24;7(10):e47939. doi: 10.1371/journal.pone.0047939 (PMC3480506; doi:10.1371/journal.pone.0047939)
Supplement: Table S2 — Criteria for quality assessment of genetic association of NQO1 gene C609T polymorphism with lung cancer. (DOC) [file pone.0047939.s002.doc]

**SUPPLEMENTARY TABLE S2.** Criteria for quality assessment of genetic association of *NQO1* gene C609T polymorphism with lung cancer

| **Criteria** | **Quality score** |
| --- | --- |
| ***Representativeness of case-patients*** | |
| 1. Consecutively/randomly selected from case population with clearly defined random frame | 2 |
| 1. Consecutive/randomly selected from case population without clearly defined random frame or with extensive inclusion criteria | 1 |
| 1. Method of selection not described | 0 |
| ***Representativeness of controls*** | |
| 1. Controls were consecutive/randomly drawn from the same area (ward/community) as cases with the same criteria | 2 |
| 1. Controls were consecutive/randomly drawn from a different area than were cases | 1 |
| 1. Not described | 0 |
| ***Ascertainment of lung cancer patients*** | |
| 1. Clearly described objective criteria for diagnosis of lung cancer | 1 |
| 1. Not described | 0 |
| ***Ascertainment of controls*** | |
| 1. Clinical examinations were performed on controls to prove that controls did not have lung cancer | 2 |
| 1. Article merely stated that controls were subjects who did not have lung cancer; no proof provided | 1 |
| 1. Not described | 0 |
| ***Ascertainment of genotyping examination*** | |
| 1. Genotyping performed under “blind” conditions | 1 |
| 1. Unblinded or not mentioned | 0 |
| ***Test for Hardy-Weinberg equilibrium*** | |
| 1. Hardy-Weinberg equilibrium in control group | 2 |
| 1. Hardy-Weinberg disequilibrium in control group | 1 |
| 1. Hardy-Weinberg equilibrium not checked | 0 |
| ***Association assessment*** | |
| 1. Assessed association between genotypes and lung cancer with appropriate statistics and adjusting for confounders | 2 |
| 1. Assessed association between genotypes and lung cancer with appropriate statistic without adjusting for confounders | 1 |
| 1. Inappropriate statistics used | 0 |
